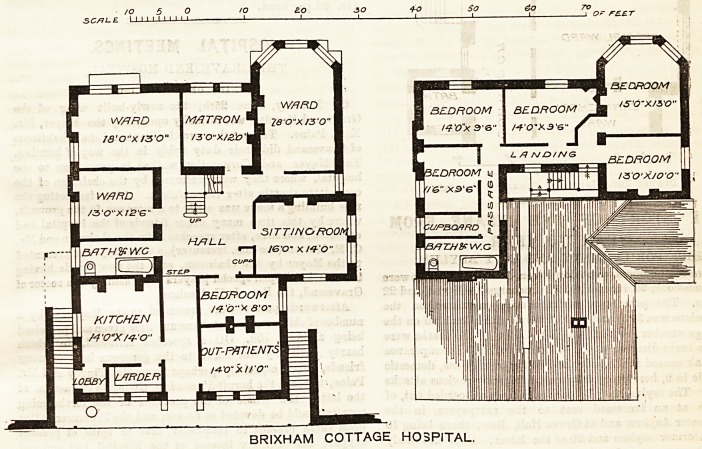# Brixham Cottage Hospital

**Published:** 1895-07-06

**Authors:** 


### The Institutional Workshop.

### HOSPITAL CONSTRUCTION.

### BRIXHAM COTTAGE HOSPITAL.

The plans we publish of this building—designed by Mr. Tollit, of Totnes, and erected at the expense of Miss Hogg as a memorial to her sister and for the benefit of the inhabitants of Brixham and its neighbourhood—show the arrangements adopted on the ground and first floors. The institution provides for nursing the poor in their own homes, as well as such as come beneath the roof of the hospital. Hence the upper floor provides sleeping accommodation for nurses largely in excess of what the ground floor seems to demand.

The wards on the ground floor are intended respectively for men, women, and children, the sitting-room is for the use of the nurses, and the room set apart for out-patients is intended also to be used for operations in cases of necessity—a decidedly unsatisfactory arrangement. A basement under a portion of the buildings provides additional larders, a scullery, and a rain-water tank.

It is impossible to regard the disposition of the rooms as a successful example of hospital arrangement, even on so small a scale as this. The positions of doors, windows, and fireplaces in the wards do not seem to have been considered in relation to the places which the beds are to occupy. The arrangement, in one apartment, of baths and w.c.'s and the direct communication of this apartment and of the sanitary appliances with the main hall of the building are, unfortunately, retrograde, and should have been avoided. The dissociation of the scullery and kitchen and the position of the latter with regard to the general entrance and the possible operating-room are not judicious arrangements. In brief, the special requirements and risks of hospital work seem to have been insufficiently considered.

BRIXHAM COTTAGE HOSPITAL.

## Figures and Tables

**Figure f1:**